# Fine Control of In Vivo Magnetic Hyperthermia Using Iron Oxide Nanoparticles with Different Coatings and Degree of Aggregation

**DOI:** 10.3390/pharmaceutics14081526

**Published:** 2022-07-22

**Authors:** Yurena Luengo, Zamira V. Díaz-Riascos, David García-Soriano, Francisco J. Teran, Emilio J. Artés-Ibáñez, Oihane Ibarrola, Álvaro Somoza, Rodolfo Miranda, Simó Schwartz, Ibane Abasolo, Gorka Salas

**Affiliations:** 1 IMDEA Nanociencia, Campus Universitario de Cantoblanco, 28049 Madrid, Spain; yurena.luengo@imdea.org (Y.L.); david.garcia.soriano@imdea.org (D.G.-S.); francisco.teran@imdea.org (F.J.T.); emilio.artes@imdea.org (E.J.A.-I.); alvaro.somoza@imdea.org (Á.S.); rodolfo.miranda@imdea.org (R.M.); 2 Drug Delivery & Targeting, Vall d’Hebron Institut de Recerca (VHIR), Universitat Autònoma de Barcelona, 08035 Barcelona, Spain; vanessa.diaz@vhir.org (Z.V.D.-R.); simo.schwartz@vhir.org (S.S.J.); 3 Functional Validation & Preclinical Research, Vall d’Hebron Institut de Recerca (VHIR), Universitat Autònoma de Barcelona, 08035 Barcelona, Spain; 4 CIBER de Bioingeniería, Biomateriales y Nanomedicina (CIBER-BBN), C/Monforte de Lemos 3-5, 28029 Madrid, Spain; 5 Unidad Asociada de Nanobiotecnología (CNB-CSIC e IMDEA Nanociencia), 28049 Madrid, Spain; 6 Biokeralty Research Institute AIE, C/Arkaute 5, 01510 Vitoria-Gasteiz, Spain; oihane.ibarrola@keralty.com

**Keywords:** magnetic hyperthermia, cancer, nanoparticles, controlled heat in vivo

## Abstract

The clinical implementation of magnetic hyperthermia has experienced little progress since the first clinical trial was completed in 2005. Some of the hurdles to overcome are the reliable production of magnetic nanoparticles with controlled properties and the control of the temperature at the target tissue in vivo. Here, forty samples of iron oxide superparamagnetic nanoparticles were prepared by similar methods and thoroughly characterized in terms of size, aggregation degree, and heating response. Selected samples were intratumorally administered in animals with subcutaneous xenografts of human pancreatic cancer. In vivo experiments showed that it is possible to control the rise in temperature by modulating the field intensity during in vivo magnetic hyperthermia protocols. The procedure does not require sophisticated materials and it can be easily implemented by researchers or practitioners working in magnetic hyperthermia therapies.

## 1. Introduction

Nanoparticles (NPs) with superparamagnetic properties are being widely studied for biomedical applications. Among other magnetic materials, iron oxide nanoparticles are by far the most studied nanomaterials for biomedicine, due to their biocompatibility and low toxicity [1]. Beyond their use as contrast agents in magnetic resonance imaging, the most distinct feature of iron oxide NPs, compared to other NPs, is their ability to release heat when they are subjected to alternating magnetic fields (AMF) [2]. Indeed, iron oxide NPs have been long proposed, alone or in combination with chemotherapeutic drugs, to treat different types of cancers through what is known as magnetic hyperthermia. Clinical trials on the subject have been conducted for almost two decades now [3]. In brief, the rationale is that the high penetration depth of magnetic fields in biological tissues allows activation of superparamagnetic NPs previously placed close to cancer cells. Under AMF, NPs will release heat, which will increase the local temperature, contributing to killing cancer cells or making them more sensitive to concomitant radio- or chemotherapy [4].

Despite the enormous efforts dedicated to the subject, just few examples of iron NP-based magnetic hyperthermia have reached clinical implementation [5,6]. The challenges that this technology faces to be applied in patients can be classified into two main groups: (i) those related to the intrinsic properties of NPs and their reliable production and (ii) those related to the interaction of NPs with biological environments and their behavior within organisms [7]. Small changes in the size and other parameters are translated into significant differences in the biological effect. Thus, production methods need to be finely tuned to minimize batch-to-batch variability and ensure biological reproducibility. In addition, those NPs dispersed in aqueous or physiological media usually undergo aggregation, which affects properties like their heating ability [8,9,10], introducing an additional variable that requires special attention.

Another important issue for the clinical translation of magnetic hyperthermia is the poor control in real-time over the temperature increase once the AMF is activated. It is widely accepted that temperatures below 40 °C degrees are not harmful to tissues [11,12] whereas cells exposed to temperatures around 42 °C will undergo cell death mainly by apoptosis. This is what is known as mild hyperthermia, and it has been demonstrated that it has beneficial effects also by provoking structural changes in the tumor microenvironment and sensitizing cancer cells to radiotherapy and/or chemotherapy [13]. However, if the temperature rises too much over 42 °C, a relatively larger number of cells will die by necrosis and irreversible damage can be caused to the surrounding healthy tissues [11,14]. One way of controlling the temperature in magnetic hyperthermia is the use of self-limiting NPs. These NPs can be designed with specific compositions that cause the deactivation of the magnetic heating when a certain specific temperature is reached, coinciding with the Curie temperature of the material [15]. However, one important limitation of this approach is that it cannot be adapted to problems that can easily arise in vivo (e.g., a lower effective concentration of the NPs at the tumor site that would require providing a higher electromagnetic dose than initially expected or calculated). Another approach is the use of hybrid NPs or nanostructures that can provide a real-time, accurate read-out of the temperature at the same time that the magnetic heating occurs. Thus, magnetic NPs have been encapsulated with luminescent NPs with optical absorption and emission in the so-called biological windows (near-infrared) to minimize absorption and dispersion by tissues. These magneto-luminescent assemblies provided in situ thermal feedback during magnetic heating [16,17]. This promising approach has, however, some limitations when considering a future implementation in clinical practice. It requires sophisticated nanomaterials and devices for the simultaneous magnetic heating and optical read-out of the temperature that will be difficult to scale up and develop under good manufacturing practices. Simple iron oxide NPs are easier to handle and produce than hybrid nanostructures. Indeed, iron oxide superparamagnetic NPs have been long approved and marketed for imaging or iron replacement therapies [18,19]. Regarding the thermal dose, for subcutaneous xenograft models, a practical way to monitor the temperature is the use of infrared thermocameras or optic fiber thermometers. Although this approach is mostly limited to the surface temperature, it has been revealed as a useful tool to understand and correlate the temperature variations with the field being applied and the amount of NPs administered. Thus, Prof. Hilger and colleagues have used thermal images (infrared thermometry) to estimate the temperature dose applied to the tumor region in xenograft models in mice with various cell lines [20,21,22,23,24]. The same approach allows recording the temperature increase and decrease when the AMF is switched on and off. Tumor surface temperature was generally maintained under 43 °C in MDA-MB-231 and BxPC-3 xenografts, corresponding to breast and pancreatic cancers, respectively. However, localized hot spots, with temperatures even higher than 45 °C, were detected in the tumor region, preferentially in MDA-MB-231 tumors [20]. Gazeau et al. also used thermal cameras to record the temperature in mice with implanted epidermoid carcinoma xenograft. Average surface temperature increases of up to 7.8 ± 2.2 °C were measured when applying an AMF of 23.8 kA/m and 111 kHz, after intratumoral injection of magnetic nanocubes (0.7 mg of iron) [25]. However, the continuous recording of temperatures in specific spots might be difficult and the time required to reach a given temperature in the tumor is only rarely reported [11]. More insight on the temperature variations would help to better estimate the amount of NPs and the field conditions necessary to reach a therapeutic effect. Together with this, a more flexible way of varying the applied AMF would also contribute to better adjusting of the temperature in real time, avoiding uncontrolled heating of the tumor, and maximizing the thermal dose to improve the therapeutic outcome.

In this work, we compare 40 iron oxide NPs samples prepared from five independent but similar syntheses by coprecipitation, followed by coating with dextran or starch to improve the colloidal stability. The structural, magnetic, and colloidal properties were studied to examine the batch-to-batch variability and its effects on magnetic heating. Even minor variations in the aggregate size give rise to significant differences in the heating ability that, in addition, depend strongly on the applied field. Selected samples were inoculated into subcutaneous tumors of MIA PaCa-2 or BxPC-3 pancreatic human cancer cells. The AMF applicator employed (MACH system, RCL) allowed adjusting the field intensity (therefore, the heat dose) in response to the temperature read-outs recorded with an optic fiber thermometer. This allowed for modulation of the AMF to get comparable results with different concentrations of NPs and with NPs from different syntheses.

## 2. Materials and Methods

### 2.1. Preparation and Characterization of the Nanoparticles

For the preparation and characterization of the nanoparticles, see details in the Supplementary Materials. Maghemite nanoparticles were obtained following a modified Massart coprecipitation protocol [26]. The so-obtained Fe_3_O_4_ nanoparticles were oxidized to maghemite (γ-Fe_2_O_3_) by a thermal acid treatment previously reported that also improves the colloidal and magnetic properties, reduces the size distribution, and activates the particle surface for further coating [27,28]. Four different batches were prepared following the previous procedure in the same conditions and with FeCl_3_·6H_2_O and FeCl_2_·4H_2_O as the iron sources (NP1, NP2, NP3, NP4). Another batch (NP0) was prepared following the same procedure but using 43 mL of a commercial solution of FeCl_3_ at 27% instead of the solid FeCl_3_·6H_2_O product. After nanoparticle synthesis, a surface modification with dextran (40 kD) or starch. Altogether, 40 samples were prepared for this work: 29 with dextran (1 of NP0, 7 of NP1, 7 of NP2, 8 of NP3, and 6 of NP4) and 11 with starch (3 of NP2 and 8 of NP3).

Prior to their use in animals, the coated magnetic nanoparticles were sterilized by gamma radiation.

Nanoparticles were characterized by different techniques: particle size and shape and size distribution were determined by transmission electron microscopy (TEM), the crystal structure of the samples was identified by X-ray powder diffraction (XRD), Fe concentration was measured with an inductively coupled plasma optical emission spectrometer (ICP-OES), hydrodynamic size and zeta potential of the particles in liquid suspensions were measured by dynamic light scattering (DLS), organic content was determined by thermogravimetry analysis (TGA), magnetic characterization was carried out in a vibrating sample magnetometer (VSM), and heating abilities of magnetic nanoparticles were evaluated through their specific absorption rates (SAR) determining the magnetic losses by AC magnetometry [29]. Details on the characterization methods and techniques are included in the Supplementary Materials.

### 2.2. Animal Experimentation

All animal experimentation was performed following procedures previously approved by the Ethical Committee for the Use of Experimental Animals (CEEA) at the Vall d’Hebron Research Institute (VHIR), Barcelona and the local government (CEA-OH/10153). In vivo studies were performed by the ICTS “NANBIOSIS”, at the CIBER-BBN’s in vivo Experimental Platform of the Functional Validation & Preclinical Research (FVPR) area (Available online: http://www.nanbiosis.es/portfolio/u20-in-vivo-experimental-platform/ (accessed on 1 May 2022) (Barcelona, Spain). Female athymic nude mice (Hsd:Athymic Nude-*Foxn1^nu^*, Envigo, Barcelona, Spain) received subcutaneous (s.c.) tumor cell injection into the right flank of exponentially growing MIA PaCa-2 or BxPC-3 pancreatic human cancer cells (10,000,000 cells per inoculum). Once tumors reached 150 mm^3^ of volume, calculated according to the formula D × d × d/2 where D is the larger diameter of the tumor and d the smaller one, NP inoculation was performed. The test items were administered per intratumoral (i.t.) route at 1 or 1.8 mg Fe/100 mm^3^ of tumor. AMF protocol was applied 2 or 24 h post-administration using a preclinical MACH system (Resonant Circuits Limited, United Kingdom). This AMF applicator allows modulating the field intensity from 4.5 to 8.0 kA/m, by adjusting the power supply voltage from 15 V to 26 V. The frequency is 1 MHz at all field intensities. Voltage was increased steadily (slow protocol), increasing 2–4 V every 3 min, or rapidly (fast protocol), going from 15 to 26 V in less than 5 min. Tumor and rectal temperature were monitored by optic fiber thermometer (Fotemp4, Optocon AG, Dresden, Germany), so voltage was maintained steady once 41 °C was reached in the tumor. AMF protocol was stopped whenever tumor temperatures reached 45 °C or after 20 min of the initiation of the protocol. Prior to animal inoculation, the heating potential of the NPs was also studied in solution with the same MACH system, keeping the voltage constant at 15 V (field intensity = 4.5 kA/m) and monitoring the temperature by immersing the optical probe into the NP solution (concentrations varying from 80.5 to 150 mg/mL in water). Up to 11 animals were used in the final experimental setting, with a minimum of 2 animals being inoculated for each nanoparticle tested.

X-ray CT images were obtained using a Quantum FX micro-CT instrument (Perkin Elmer, Waltham, MA, USA). Incident X-ray tube voltage was set at 50 kVp and amperage at 160 μA. Acquisition time was 4.5 min. Field of view was 30 mm, corresponding to 0.059 mm spatial resolution. CT scans were performed 24 h post-injection. Reconstruction of the studies was performed with the Quantum FX software and final images were processed with Amide software version 1.0.2 [30].

## 3. Results and Discussion

### 3.1. Characterization of the Nanoparticles

#### 3.1.1. Characterization of Uncoated Nanoparticles (NPs)

**Figure 1:**
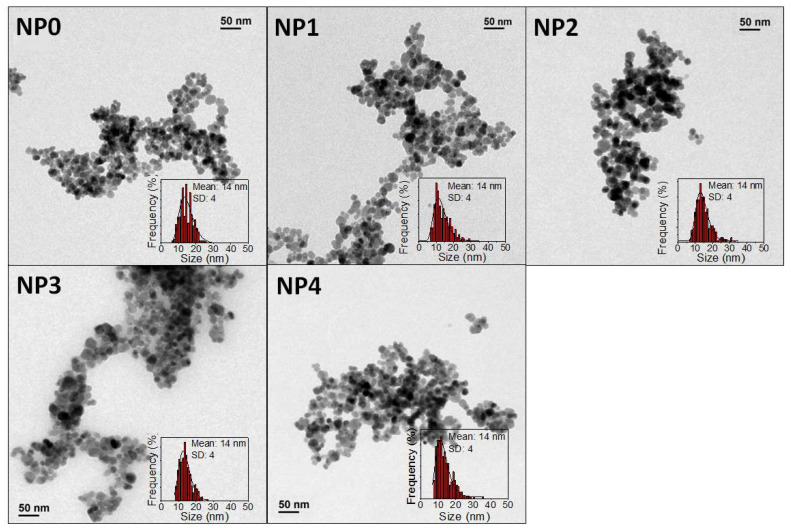
TEM micrographs and size distribution of different coprecipitation samples.

Firstly, the morphological properties (size and shape) as well as the particle size distribution of the uncoated samples were studied. Five different batches were prepared from five independent reactions following the same experimental procedure. After inspecting the samples by means of TEM, we found nearly spherical uniform NPs with mean diameters (D_TEM_) of 14 ± 4 nm in all cases (Figure 1 and Table 1).

The structural and magnetic properties of uncoated NPs were also characterized. Figure S1A shows the X-ray diffractograms for the five different batches prepared. XRD has been used both to identify the nature of the material and to determine the particle size. All the peaks are ascribed to a spinel structure, most probably maghemite (γ-Fe_2_O_3_). The width of the peaks in the diffractogram is related to the NP crystal size. Using the Scherrer equation for the width values obtained from the main (311) peak crystal sizes of 16.1, 16.7, 15.5, 16.9, and 15.7 nm are obtained for NP0, NP1, NP2, NP3, and NP4 samples, respectively. The similitude of XRD and TEM size confirms the monocrystalline nature of the samples.

Magnetization curves of uncoated samples in powder form at room temperature (Figure S1B) showed superparamagnetic behavior with negligible coercivity and remanent magnetization. The saturation magnetization values (66, 73, 75, 74, and 77 A·m^2^/kg for NP0, NP1, NP2, NP3, and NP4 samples, respectively) are very similar in all cases and close to the bulk value [31], which is related with the high crystallinity of the samples.

Considered together, TEM, XRD, and VSM results confirm the high reproducibility of the coprecipitation synthesis method.

#### 3.1.2. Characterization of Dextran and Starch Coated NPs

**Figure 2:**
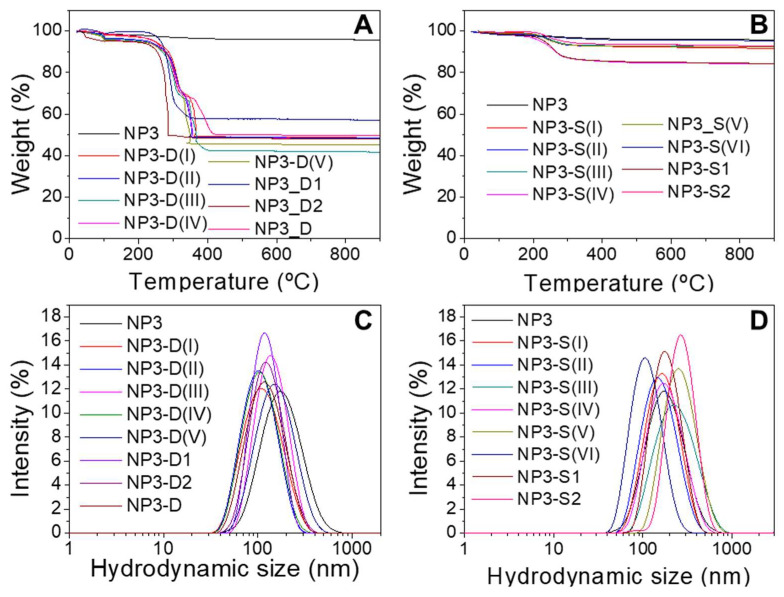
Thermogravimetric analysis (TGA) of NP3 samples coated with dextran (**A**) and starch (**B**). Hydrodynamic size distribution of NP3 samples coated with dextran (**C**) and starch (**D**). Each line corresponds to a single batch prepared under the same conditions.

Different batches of dextran and starch coated maghemite NPs were prepared using the five samples previously synthesized. Dextran and starch are carbohydrates very commonly used as coatings for nanoparticles because of their biocompatibility [32,33]. The coating process was carried out by keeping the sample in an ultrasonic bath for 10 h so that the effectiveness of the coating molecules’ union to the NP surface, as well as the final NP dispersion, could present certain variability depending on how the sample receives the ultrasonic waves. TGA allowed determining the percentage organic content of the nanoparticles, due to the coating. Figure 2 shows analysis of NP3 without coating and coated with dextran (Figure 2A) and starch (Figure 2B). In all cases, a greater weight loss can be observed for samples coated with dextran than uncoated ones because of the carbohydrates bound to the NPs. Similar results are obtained for NP0, NP1, NP2, and NP4 coated samples (Figure S2).

Samples with starch display organic contents around 5% and 15% lower than in the case of dextran-coated NPs, ~40–50% of weight loss when no ultracentrifugation was employed during the work-up of the process. When the nanoparticles were subjected to ultracentrifugation through 100 kDa filters (NP4 samples), lower weight losses of around 15–35% were obtained (Figure S2D). This centrifugation process removes dextran molecules that are not strongly bound to the NPs. It is worth noting, however, that this purification step does not seem to be related to the variations observed in hydrodynamic sizes, the magnetism, or the heating abilities of the resulting coated NPs, as will be discussed below.

When aggregation of NPs occurs, the hydrodynamic diameters (D_hyd_) measured by DLS are a suitable parameter to quantify the aggregate size [10]. The aggregate size distributions, based on intensity, of NP3 sample before and after coating with dextran and starch are shown in Figure 2. It can be seen that a mono-modal size distribution for each sample appears in all cases. Samples coated with dextran present smaller hydrodynamic sizes than the corresponding starch-coated samples (D_hyd_ values vary from 79 to 140 nm for dextran-coated samples and from 106 to 287 nm for starch-coated samples), which indicates a lesser aggregation degree in water and therefore less interparticle interaction. This trend is observed in general for all the samples studied (Figure S3) and is also apparent from the TEM micrographs of coated NPs (Figure S4). To control the aggregation degree of the NPs during the coating process, it is crucial to control the ultrasounds bath temperature and the homogeneity of the sonication process, which implies always using the same recipient with the same volume. Further improvements for controlling the aggregate size could imply size-sorting procedures by centrifugation, ultracentrifugation, or filtration through filters with appropriate pore sizes. The Z-potential at pH 7 has also been measured to determine the NP surface charge. Both dextran and starch have only –OH groups, which can be protonated or deprotonated, so the surface charge is not very high (between 15 and −15 mV). These carbohydrates are macromolecules that provide steric hindrance and, thus, colloidal stability [32,33].

Finally, the magnetic characterization of the coated samples confirms that the coatings do not affect the magnetic behavior (Figures S5 and S6). A summary of all the characterization data is provided on Table S1.

#### 3.1.3. Influence of Aggregation on the SAR Values

**Figure 3:**
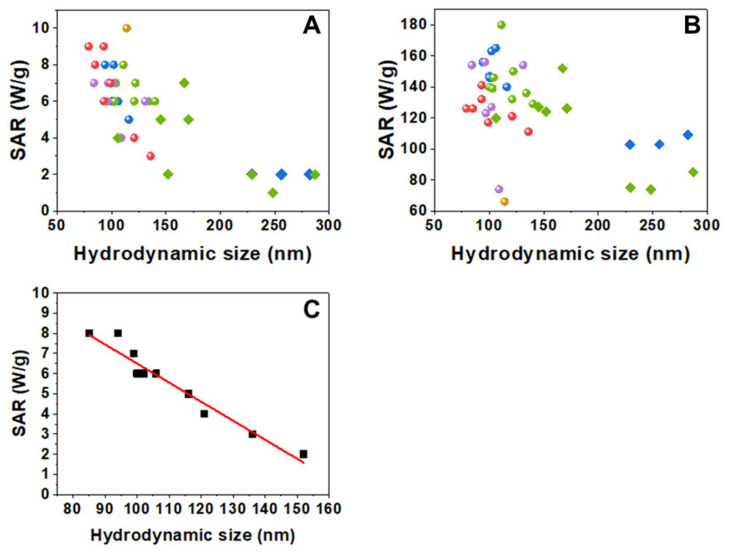
SAR values vs. hydrodynamic size measured at two different field conditions: (**A**) 300 kHz, 4 kA/m and (**B**) 100 kHz, 24 kA/m. Circles correspond to the dextran-coated samples and diamonds to the starch-coated samples (yellow for NP0, red for NP1, blue for NP2, green for NP3, and purple for NP4 batches). (**C**) SAR values vs. hydrodynamic size of nanoparticles with D_hyd_ < 155 nm and with PdI ≤ 0.15, measured at 300 kHz, 4 kA/m. The fit shows a linear decrease of SAR with D_hyd_ (slope = −0.09 W·g^−1^·nm^−1^, intercept = 15.9 W·g^−1^, R^2^ = 0.94).

The heating abilities of dextran and starch coated samples were evaluated through their specific absorption rate (SAR) values and measured at different field and frequency conditions (4 kA/m, 300 kHz and 24 kA/m, 100 kHz). For each nanoparticle sample, higher SAR values were obtained at the higher field intensity conditions, as expected (Table S1 and Figure 3). It is known that aggregation usually causes a reduction of the heat released by magnetic NPs and that even small changes in the degree of aggregation may lead to changes in the heating ability of the nanoparticles [9,10]. In addition, that SAR reduction will also depend on the applied AC field conditions. Figure 3 shows SAR values as a function of aggregate size (D_hyd_ values). These data also allow comparison of the batch-to-batch variability of the forty samples studied obtained from the five different core syntheses and the subsequent coatings for each of them. Altogether, there is a clear general trend of the SAR to decrease as the hydrodynamic size increases. The calorimetric measurements show a rather dispersed variation of SAR values with the hydrodynamic size that, in addition, depend strongly on the conditions of the applied field (Figure 3), with the decrease being more similar in appearance to an exponential decay under 300 kHz and 4 kA/m. This lowering in the heating ability as the aggregation increases can be explained by considering the detrimental effect of interparticle dipolar interactions within the aggregates [34,35,36,37]. Moreover, the SAR values are influenced not only by the size of the aggregate (and the number of nanoparticles per aggregate) but also by their polydispersity [9]. If we consider only those nanoparticles’ batches that have D_hyd_ values around 150 nm or lower, and with very narrow size distributions (PdI ≤ 0.15), SAR values diminish linearly with D_hyd_ (Figure 3).

In summary, D_TEM_ was the same in all syntheses, while D_hyd_, Z-potential, M(H), and TGA results show non-negligible variations between batches, even if the samples were prepared following the same methods and using the same reagents. Nevertheless, that variability is relatively small when compared with a previous report specifically studying batch-to-batch variability in nanoparticulate systems of SiO_2_, ZnO, CeO_2_, and TiO_2_ [38]. However, these small variations do have an important effect on the heating process. The hydrodynamic size has been reported as one of the parameters that have a strong influence on the heating ability of superparamagnetic nanoparticles even in systems apparently similar obtained from commercial sources. Thus, variation of SAR values of 30% has been reported with just three seemingly identical samples [39]. In our case, an extensive study of 40 samples shows that the hydrodynamic size plays a major role in SAR variations, and it is the most important parameter when comparing samples with low D_hyd_ and PdI values. The different types of washings that have been carried out reduce the amount of dextran but do not seem to be related to the variations in SAR values.

### 3.2. In Vivo Hyperthermia Experiments

**Figure 4:**
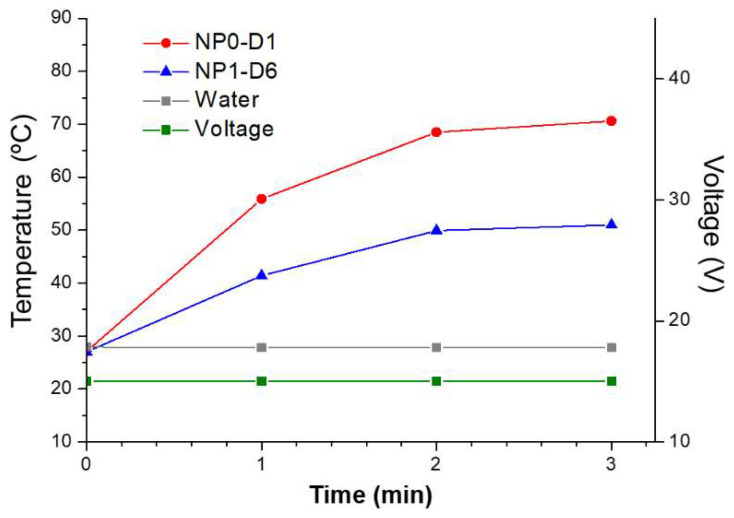
Heating abilities of NP0-D1 (red circles) and NP1-D6 (blue triangles) nanoparticles dispersed in water using the MACH system, keeping voltage fixed at 15 V (green line), corresponding to a field intensity of 4.5 kA/m. Results show that the nanoparticles increased the temperature of the media rapidly, even at the lowest voltage. Without nanoparticles, no temperature increase was detected (grey squares).

Prior to their use in vivo, samples were sterilized under gamma irradiation. NP0-D1, NP1-D6, NP2-D6, NP3-D8, and NP4-D3 were selected for testing the effect of magnetic hyperthermia against pancreatic tumors in mice. These nanoparticles had shown relatively high SAR values under the field intensity of 4 kA/m (close to the MACH system field intensities) and fairly low D_hyd_ between 90 and 110 nm. Among them, NP0-D1 and NP1-D6 were the samples with the highest SAR values (10 W/g and 9 W/g, respectively) under the AMF of 300 kHz and 4 kA/m. When aqueous samples of NP0-D1 and NP1-D6 are subjected to a constant field of 4.5 kA/m, both samples increase the temperature of the medium above 40 degrees in less than 1 min (Figure 4). After 3 min, the medium reaches 70 and 50 °C for samples NP0-D1 and NP1-D6, respectively. This difference may be because the concentration of the NP0-D1 sample is slightly higher. If the field intensity is gradually increased from 4.5 kA/m (14 V) to 8.0 kA/m (26 V) the medium eventually reaches 100 °C (Figure S7). If pure water is subjected to the same AMF conditions, no temperature increase can be detected, indicating that the MACH applicator is not inducing any heating in the absence of nanoparticles under the studied conditions.

**Figure 5:**
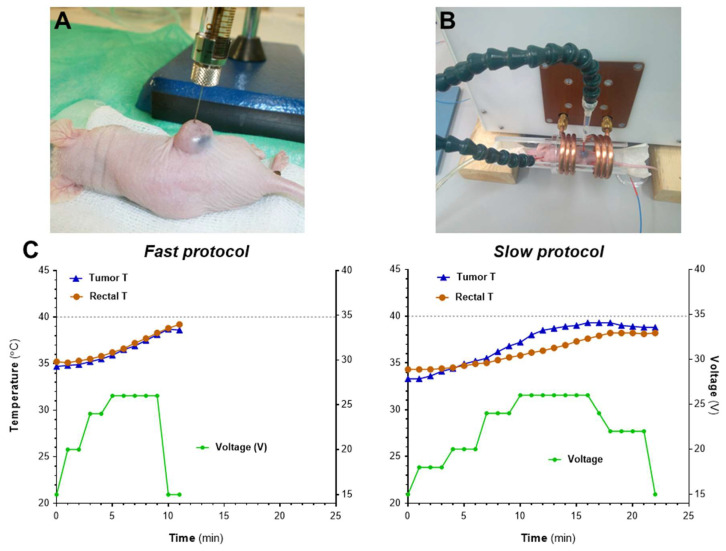
In vivo optimization of heating protocols using the MACH system. (**A**) Mice bearing s.c. MIA PaCa-2 tumors were inoculated i.t. with NP2-D6 (1 mg Fe/100 mm^3^). (**B**) Twenty-four hours after administration, mice (*n* = 2 protocol) were exposed to AMF and body and tumor temperatures were recorded by two optical thermal probes. (**C**) AMF was applied using two different protocols: fast increase of voltage (left graph, *fast protocol*) or slow and steady increase in voltage (right graph, *slow protocol*). Graphs show one representative example of each procedure.

Once the heating of the samples in water had been verified, an in vivo optimization of the heating protocol using the MACH system was carried out (Figure 5). We monitored the rectal temperature and the temperature at the tumor site when field intensity was gradually increased at 2 different rates (*fast* and *slow* protocols). An increase in the tumor temperature was observed in both cases, although higher values were achieved with the slow protocol. Significantly, the use of the fast protocol induced a rapid increase of the overall body temperature in the mice as measured by the rectal probe, inducing the early end of the experiment when rectal temperatures reached 39 °C. The slow protocol provides a steady increase of the tumor temperature without increasing the rectal temperature too much (Figure 5C); this protocol was implemented in further assays. It is worth noting that tumor temperatures would be probably higher than the ones recorded by the probe, since the probe only measures one specific point in the tumor and that might not be reflective of the overall tumor temperature inside, nor that of any “hotspot” in the tumor.

**Figure 6:**
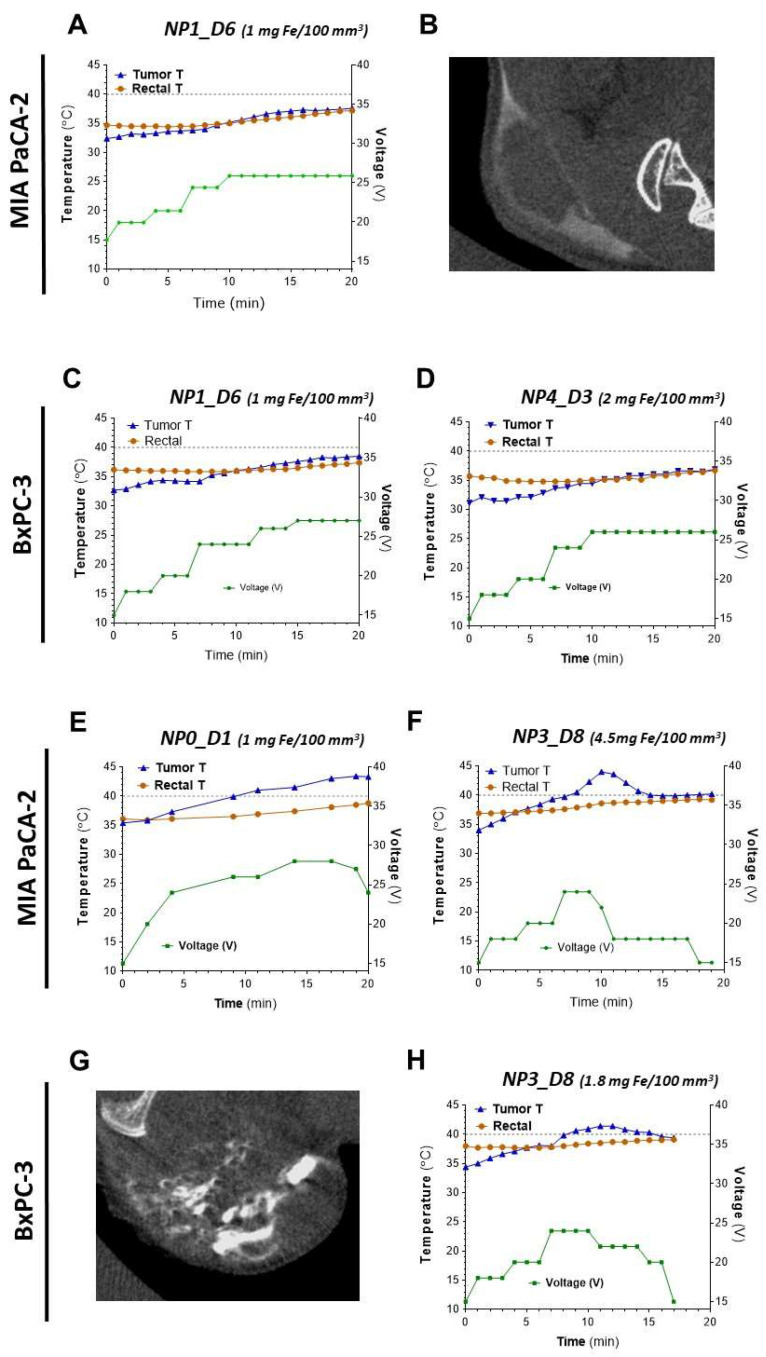
Heating abilities and intratumoral distribution of NP0-D1 to NP4-D3 in s.c. pancreatic MIA PaCa-2 and BxPC3 tumors. Concentrations express the amount of nanoparticles (in mg of iron) per 100 mm^3^ of tumor volume. (**A**–**D**) NP1-D6 and NP4-D3, (**E**–**H**) NP0-D1 and NP3-D8. The variations with time of the recorded temperature at the tumor site (in blue), the rectal temperature (in orange), and the applied voltage (in green) are shown for the indicated samples (**A**,**C**–**F**,**H**). The grey dashed line represents the threshold of 40 °C. Intratumoral biodistribution of nanoparticles was imaged by CT for NP1-D6 and NP3-D8 (**B** and **G**, respectively). Only in tumors inoculated with NP0-D1 and NP3-D8 temperatures above 40 °C were recorded.

Figure 6 shows examples of the temperature changes induced by magnetic heating and the intratumoral distribution of the studied samples. Temperatures of control animals without NP inoculation in the presence or absence of the magnetic field were also recorded (Figure S8). As for the animals with NP inoculation, it can be seen that sample NP1-D6 present a very similar behavior with both MIA PaCA-2 and BxPC-3 tumors (Figure 6A,E), without reaching 40 °C in any case. Sample NP0-D1 in MIA PaCA-2 reaches a higher temperature than NP1-D6 at the same concentration of nanoparticles dosed per tumor volume of 1 mg Fe/100 mm^3^ (Figure 6A,C). NP0-D1 was the sample that exhibited the highest SAR value in water under 4 kA/m and 300 kHz (10 W/g). The 40 °C threshold, considered as a “safety temperature” [11], was overpassed only with NP0-D1 and NP3-D8. The latter had shown a lower SAR value in water (8 W/g) than NP0-D1 and it was necessary to increase the concentration of nanoparticles (1.8 to 4.5 mg of Fe per 100 mm^3^. Figure 6D,H) to reach that temperature. Notably, with NP4-D3, it was not possible to reach 40 °C despite dosing a concentration of 2 mg Fe/100 mm^3^ (Figure 6F). Among the samples employed for the in vivo experiments, NP4-D3 had the lowest SAR value in water (7 W/g, 4 kA/m, and 300 kHz) and a high PdI (0.22). In addition, CT images show a better distribution of the nanoparticles along the tumor region with NP3-D8 (Figure 6G) than with NP1-D6 (Figure 6B). NP3-D8 sample was prepared with the purifying step of ultracentrifugation, instead of a simple dialysis as with NP1-D6, which removed most of the dextran molecules that were not tightly bound to the nanoparticles. This fact could affect the distribution of nanoparticles within the tumor.

In all cases, the increase in the temperature observed in the animals is due to the presence of the iron oxide NPs and the application of the magnetic field, as rectal temperature in animals without NP inoculation did not increase significantly (Figure S8). However, it is clearly observable that rectal temperature increases with the time that animals are subjected to the magnetic field. The source of such basal heating is not clear; it might be the result of the combination of several factors, including the dispersion of the heating from the tumor, heating of NP that might have reached the circulation (and thus other parts of the body), and the dielectric heating of the animal independent of the NPs.

An interesting and useful feature of the tumor temperature readouts is that they are clearly correlated with the voltage applied (field intensity), with a time delay of a few minutes. This can be observed in all graphs in Figure 6. For example, in Figure 6D, the change in voltage from 20 V (6.2 kA/m) in minute 6 to 24 V (7.4 kA/m) in minute 7, and then lowering it down to 18 V (5.5 kA/m) in minute 11, is followed by an increase in the tumor temperature with a delay of 2–3 min from ~40 °C to 44 °C (the maximum temperature reached in the experiment) and then a decrease to 40 °C again in minute 14. A similar behavior, with the tumor temperature rising after 2–3 min of increasing the voltage applied and decreasing after the voltage is lowered, can be observed in all experiments. The difficulty in controlling the rise of the temperature has been accurately described as one of the major drawbacks for the application of magnetic hyperthermia [11]. It is also observable in our results and also in the literature that variability in the heating rates of the tumors is high. The heterogeneity of the tumors and the inhomogeneous distribution of the NPs already observed in the CT images, as well as the difficulties to record temperature hot-spots by a surface probe, may account for such variability. In any case, our results show that adjusting the field intensity as a response to the thermal read-outs can be a practical and affordable way of obtaining 2–3 min leeway to adjust the temperature in magnetic hyperthermia protocols.

## 4. Conclusions

Maghemite NPs with different coatings were prepared following the same synthetic procedures. The obtained NPs had identical core sizes but differed in their degree of aggregation in water, probably due to small changes during their coating process. This allowed for studying the influence of the hydrodynamic size on the SAR in samples that were otherwise expected to be very similar. We found that the batch-to-batch variability of the samples was relatively small if compared to the scarce existing studies. Nevertheless, these small variations do have a significant effect on the magnetic heating abilities, especially the variations found in the hydrodynamic size, which are related to the aggregation of the nanoparticles. The different types of washings during the coating process do not seem to be related to the variations in SAR values, but they do affect the distribution of the nanoparticles in the tumor due to differences in the amount of dextran on the nanoparticles.

In vivo magnetic hyperthermia studies demonstrate the usefulness and versatility of being able to modulate the field intensity in response to the thermal read-out. The slow protocol worked better than fast protocol, providing a steady increase of the tumor temperature without increasing the rectal (systemic) temperature of the mice too much. The maximum temperature that can be reached in the tumor depends on the properties of the nanoparticles, their concentration, the field applied, and the nature of the tumor itself. Higher heating is observed in BxPC3 than in MIA PaCa-2 treated with the same sample. Finally, our results adjusting the field intensity as a response to the thermal read-outs are a practical and affordable way of obtaining 2–3 min leeway to adjust the temperature.

## Figures and Tables

**Table 1:** Labels used for each batch of nanoparticles before and after coating with dextran or starch.

Uncoated Cores	Dextran Coating	Starch Coating
NP0	NP0-D1	-
NP1	NP1-D1, NP1-D2, NP1-D3, NP1-D4, NP1-D5, NP1-D6, NP1-D7	-
NP2	NP2-D1, NP2-D2, NP2-D3, NP2-D4, NP2-D5, NP2-D6, NP2-D7	NP2-S1, NP2-S2, NP2-S3
NP3	NP3-D1, NP3-D2, NP3-D3, NP3-D4, NP3-D5, NP3-D6, NP3-D7, NP3-D8	NP3-S1, NP3-S2, NP3-S3, NP3-S4, NP3-S5, NP3-S6, NP3-S7, NP3-S8
NP4	NP4-D1, NP4-D2, NP4-D3, NP4-D4, NP4-D5, NP4-D6	-

## Data Availability

Not applicable.

## Supplementary Materials

The following supporting information can be downloaded at: https://www.mdpi.com/article/10.3390/pharmaceutics14081526/s1, details on the synthesis, coating and characterization of the nanoparticles; Figure S1: X-ray diffraction patterns and M(H) loops of uncoated samples; Figure S2: thermogravimetric analyses; Figure S3: hydrodynamic size distributions of coated nanoparticles; Figure S4: additional TEM micrographs; Figure S5: M(H) loops of dextran-coated samples; Figure S6: M(H) loops of starch-coated samples; Figure S7: temperature increase of NP4-D3 nanoparticles dispersed in water using the MACH system and increasing the voltage gradually; Table S1: summary of the characterization data. Figure S8: temperature and voltage recordings in animals without NP inoculation.
