# Supplementary material for: Fine Control of In Vivo Magnetic Hyperthermia Using Iron Oxide Nanoparticles with Different Coatings and Degree of Aggregation
Source: Pharmaceutics. 2022 Jul 22;14(8):1526. doi: 10.3390/pharmaceutics14081526 (PMC9331462; doi:10.3390/pharmaceutics14081526)
Supplement: Supplementary file 1 [file pharmaceutics-14-01526-s001.zip › pharmaceutics-1762752-supplementary.pdf]

Supplementary Material for

Fine control of in vivo magnetic hyperthermia using iron oxide nanoparticles with different coatings and degree of aggregation

Yurena Luengo,[a] Zamira V. Díaz-Riascos,[b,c,d] David García-Soriano,[a] Francisco J. Teran,[a,e] Emilio J. Artés-Ibáñez,[a] Oihane Ibarrola,[f] Álvaro Somoza,[a,e] Rodolfo Miranda,[a,e] Simó Schwartz Jr.,[b,d] Ibane Abasolo,[b,c,d], Gorka Salas[a,e]

[a] IMDEA Nanociencia, Campus Universitario de Cantoblanco, 28049 Madrid, Spain.

[b] Drug Delivery & Targeting CIBBIM-Nanomedicine, Vall d'Hebron Institut de Recerca (VHIR), Universitat Autònoma de Barcelona, 08035 Barcelona, Spain.

[c] Functional Validation & Preclinical Research, CIBBIM-Nanomedicine, Vall d'Hebron Institut de Recerca (VHIR), Universitat Autònoma de Barcelona, 08035 Barcelona, Spain.

[d] CIBER de Bioingeniería, Biomateriales y Nanomedicina (CIBER-BBN), C/ Monforte de Lemos 3-5, 28029 Madrid, Spain.

[e] Unidad Asociada de Nanobiotecnología (CNB-CSIC e IMDEA Nanociencia), 28049 Madrid, Spain.

[f] Biokeralty Research Institute AIE, C/Arkaute 5, 01510 Vitoria-Gasteiz, Spain.

Materials and Methods

Materials.

Iron(III) chloride hexahydrate (97% Aldrich), iron(II) chloride tetrahydrate (≥ 99:0%, Fluka), iron (III) chloride technical aqueous solution (27% VWR), ammonium hydroxide solution (25% in water, Fluka), iron(III) nitrate nonahydrate (≥ 98:0%, Fluka), dextran (from Leuconostoc spp., Mr ~ 40,000, Sigma), hydroxyethyl starch (Aldrich), were used as received. Amicon ultra centrifugal filters were Ultracel-100 K (Merck).

Synthesis of maghemite nanoparticles.

Maghemite nanoparticles were obtained following a modified Massart coprecipitation protocol.[1] Briefly, magnetite (Fe_3O_4) nanoparticles were synthesized by adding, drop by drop at a 0.1 mL/s rate, 75 mL of NH_4OH (25 %) solution to 425 mL of an aqueous solution of $FeCl_3 \cdot 6H_2O$ (24.3 g, 0.09 mol) and $FeCl_2 \cdot 4H_2O$ (10.7 g, 0.054 mol). To increase the particle size, a subsequent thermal treatment at 90 °C for 3 h was carried out. After every synthesis, the particles were washed three times with distilled water and collected with the help of a permanent magnet. Then, the Fe_3O_4 nanoparticles were oxidized to maghemite (γ-Fe_2O_3) by a thermal acid treatment previously reported that also improves the colloidal and magnetic properties, reduces the size distribution to a polydispersity degree of around 0.2, and activates the particle surface for further coating.[2,3] Briefly, 300 mL of HNO_3 (2 M) was added to 500 mL of the dispersion produced previously, and the mixture was stirred for 15 min. Then, the supernatant was removed by magnetic decantation and 75 mL of $Fe(NO_3)_3$ (1 M) and 130 mL of water were added to the particles. The mixture was heated to boiling temperature and stirred for 30 min. The particles were then cooled to room temperature and, by magnetic decantation, the supernatant was substituted by 300 mL of HNO_3 (2 M) and the solution stirred for 15 min. Finally, the particles were washed three times with water. Four different batches were prepared following the previous procedure in the same conditions (NP1, NP2, NP3, NP4). Another batch (NP0) was prepared following the same procedure but using 43 ml of a commercial solution of $FeCl_3$ at 27% instead of the solid product.

Coating with dextran and starch.

After nanoparticle synthesis, a surface modification with dextran (40 kD) or starch was carried out in a thermoregulated ultrasonic bath. 800 mg of polysaccharide dissolved in 10 mL of distilled water

were added to a dispersion of 800 mg γ-Fe_2O_3 in 2 mL of 2.5 M NaOH. The mixture was sonicated for 10 h at 20 ºC and washed by either dialysis (dextran-coated NP0, NP1, NP2 and NP3), centrifugation using ultra centrifugal filters of 100 kDa (dextran-coated NP4) or centrifugation (starch-coated NP2 and NP3), Then, the pH of all the samples was adjusted to 7. Altogether, 40 samples were prepared for this work: 29 with dextran (1 of NP0, 7 of NP1, 7 of NP2, 8 of NP3 and 6 of NP4) and 11 with starch (3 of NP2 and 8 of NP3).

Coated magnetic nanoparticles were sterilized by gamma sterilization. Post-production gamma sterilization was performed with up to 25 vials packaged per 49-well Cryobox container, and with no two vials placed in adjacent wells (so as to avoid overshadowing effects). Gamma sterilization conditions were: radiation source: Cobalt-60; Gamma ray energies: 1.17 MeV and 1.33 MeV; specified dosage range: from 25 kGy to 40 kGy.

Characterization.

Particle size, shape and size distribution were determined by transmission electron microscopy (TEM) using a JEOL JEM-1010 microscope operated at 100 keV and equipped with a Gatan Orius 200 SC digital camera. TEM samples were prepared by placing one drop of a dilute suspension of maghemite nanoparticles in water on a carbon-coated copper grid and allowing the solvent to evaporate at room temperature. The size distributions were evaluated by measuring the largest internal dimension of at least 300 particles found in randomly selected areas of the micrographs, with ImageJ software to obtain the mean size and standard deviation.

The crystal structure of the samples was identified by X-ray powder diffraction (XRD) performed in a Bruker D8 Advance powder diffractometer using Cu Kα radiation with an energy-discriminator (Sol-X) detector. The patterns were collected between 20º and 70º in 2θ. The XRD spectra were indexed to an inverse spinel structure. The average crystallite size was calculated by Scherrer's equation using the half width of the (311) X-ray diffraction peak using the utilities of the automatic powder diffraction computer program X'Pert HighScore from Malvern Panalytical. The error in the crystallite sizes obtained by use of the Scherrer's equation is ± 0.1 nm and is related to the instrumental line width of the diffractometer ($\Delta 2\theta = 0.11^{\circ}$).

The Fe concentration was measured with an inductively coupled plasma optical emission spectrometer (ICP-OES) Perkin-Elmer Optima 2100 DV. For this purpose samples were digested with hydrochloric acid to dissolve the particles.

Colloidal properties of the samples were studied in a Zetasizer Nano ZS, from Malvern Instruments. The hydrodynamic size of the particles in suspensions was measured by dynamic light scattering (DLS) from dilute suspensions of the sample in water at pH 7 in a standard cuvette. The energy source was a laser emitting red light, and the angle between the sample and detector is 173º. Hydrodynamic size in this work will refer to the Z-average size in intensity, since this is the most reliable value produced by the DLS technique, the size distribution was evaluated from the polydispersity index (PdI), and the zeta potential was measured at pH 7 using 10^{-2} M KNO_3 as background electrolyte.

The organic content was determined by thermogravimetry analysis (TGA). The measurements were performed by using a TGA Q500 instrument (TA Instruments) at a heating rate of 10 ºC/min under an air atmosphere, from room temperature up to 900 ºC.

The magnetic characterization of the magnetic nanoparticles was carried out before and after coating in a vibrating sample magnetometer (Lakeshore, Oxford Instrument) at room temperature by first saturating the samples in a field of 2 T. Samples were measured in solid state. For the measurement of powders, the samples were dried in an inox-coated oven at 50 ºC 24 h. Afterward samples were accurately weighed and fitted into the gelatin sample holder. The saturation magnetization was evaluated by extrapolating to infinite field the experimental data obtained in the field range where magnetization increases and it can be approximated to a 1/H law. Magnetization is expressed in $A \cdot m^2$ per kilogram of maghemite, taking into account the inorganic/organic ratio obtained from TGA analyses.

The heating abilities of magnetic nanoparticles were evaluated through determining the magnetic losses by AC magnetometry at two different fields (100 kHz and 24 kA/m or 300 kHz and 4 kA/m). For that purpose, a home-made inductive magnetometer set up similar to the one described by Connord *et al*. was employed.[4] The system quantifies the specific absorption rate (SAR) values from AC hysteresis loops considering the expression (Eq. 1):

$$SAR = A \cdot f \qquad (1)$$

where *A* is the area of the hysteresis loop under alternating magnetic fields and *f* is the magnetic field frequency. Magnetization units were normalized by the iron mass and expressed in $A \cdot m^2/kg_{Fe}$. Each AC hysteresis loop was obtained from three repetitions, resulting in the averaged magnetization cycle and the related magnetic parameters: coercive field (H_C), remanent magnetization (M_R), and the area of the hysteresis loop.

References

[1] R. Massart, Preparation of aqueous magnetic liquids in alkaline and acidic media, IEEE Trans. Magn. 17 (1981) 1247–1248. https://doi.org/10.1109/TNB.2008.2005325.

[2] R. Massart, E. Dubois, V. Cabuil, E. Hasmonay, Preparation and properties of monodisperse magnetic fluids, J. Magn. Magn. Mater. 149 (1995) 1–5. https://doi.org/10.1002/jcb.23384.

[3] R. Costo, V. Bello, C. Robic, M. Port, J.F. Marco, M.P. Morales, S. Veintemillas-Verdaguer, Ultrasmall iron oxide nanoparticles for biomedical applications: improving the colloidal and magnetic properties., Langmuir. 28 (2012) 178–85. https://doi.org/10.1021/la203428z.

[4] V. Connord, B. Mehdaoui, R.P. Tan, J. Carrey, M. Respaud, An air-cooled Litz wire coil for measuring the high frequency hysteresis loops of magnetic samples - A useful setup for magnetic hyperthermia applications, Rev. Sci. Instrum. 85 (2014) 093904. https://doi.org/10.1063/1.4895656.

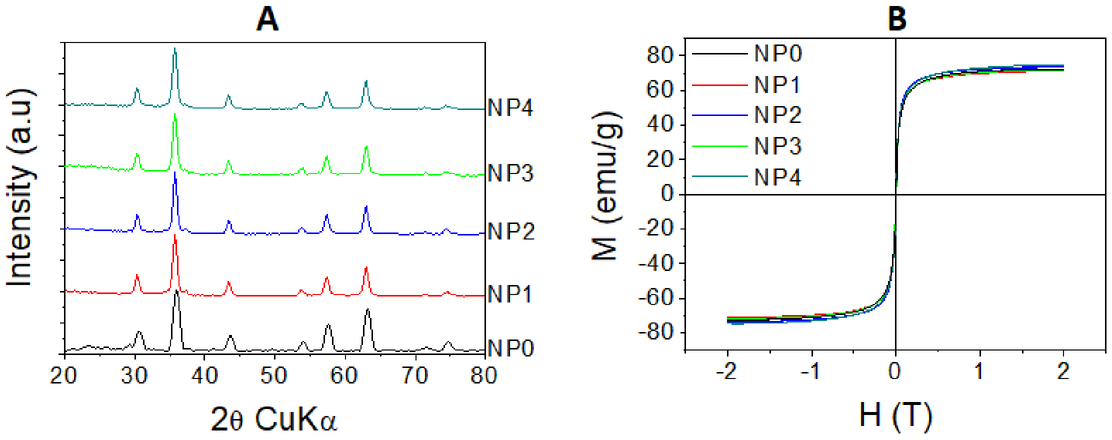

Figure S1. X-ray diffraction patterns (A) and hysteresis loops (B) of uncoated samples NP0, NP1, NP2, NP3 and NP4.

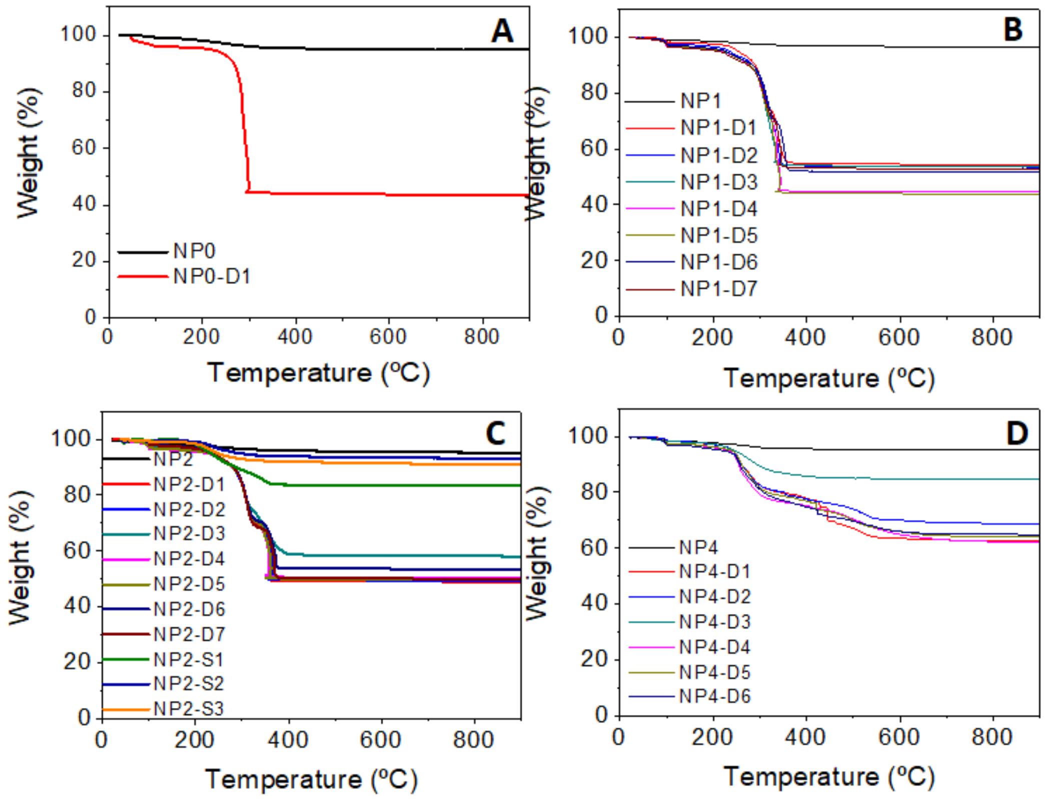

Figure S2. TGA results NP0 (A), NP1 (B), NP2 (C) and NP3 (D) coated with dextran and starch.

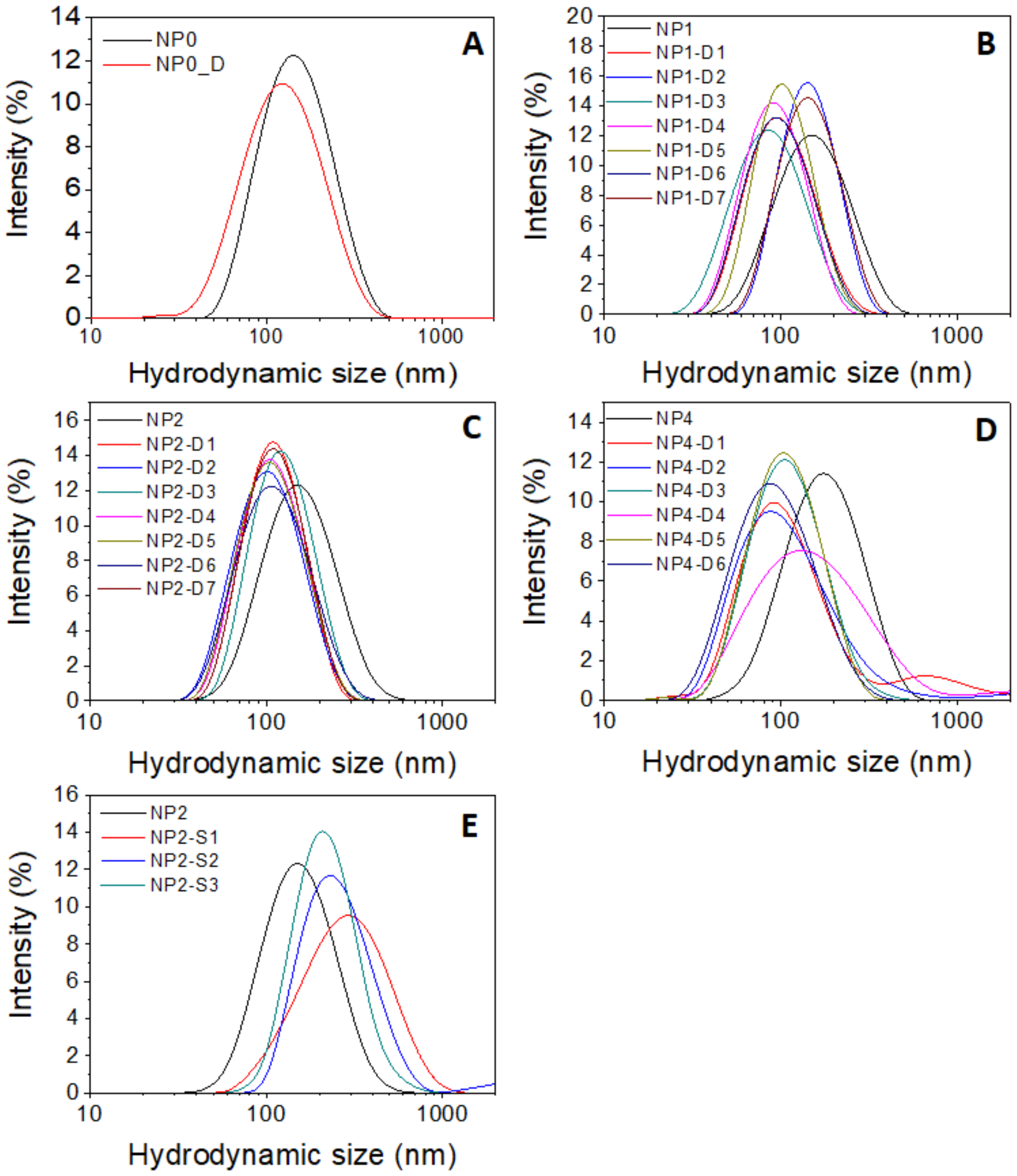

Figure S3. Hydrodynamic size distribution of dextran coated NP0 (A), NP1 (B), NP2 (C) and NP3 (D) and starch coated NP2 (E) samples.

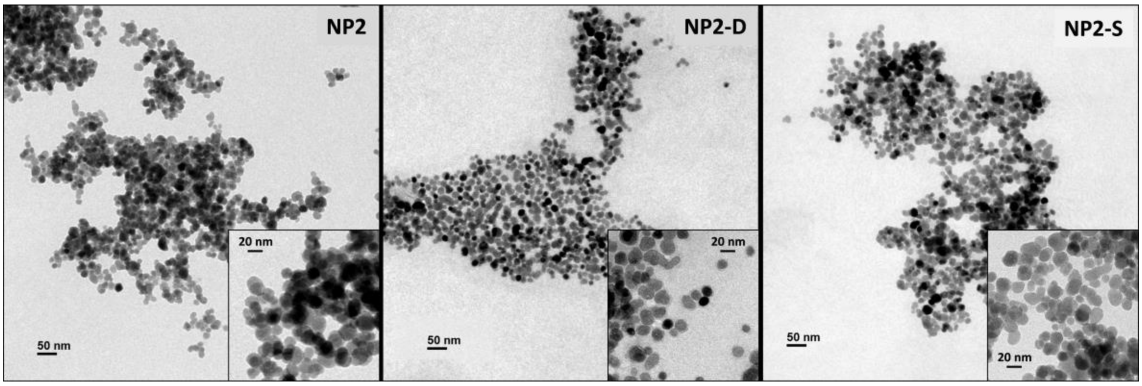

Figure S4. TEM micrographs of NP2 sample uncoated and coated with dextran and starch.

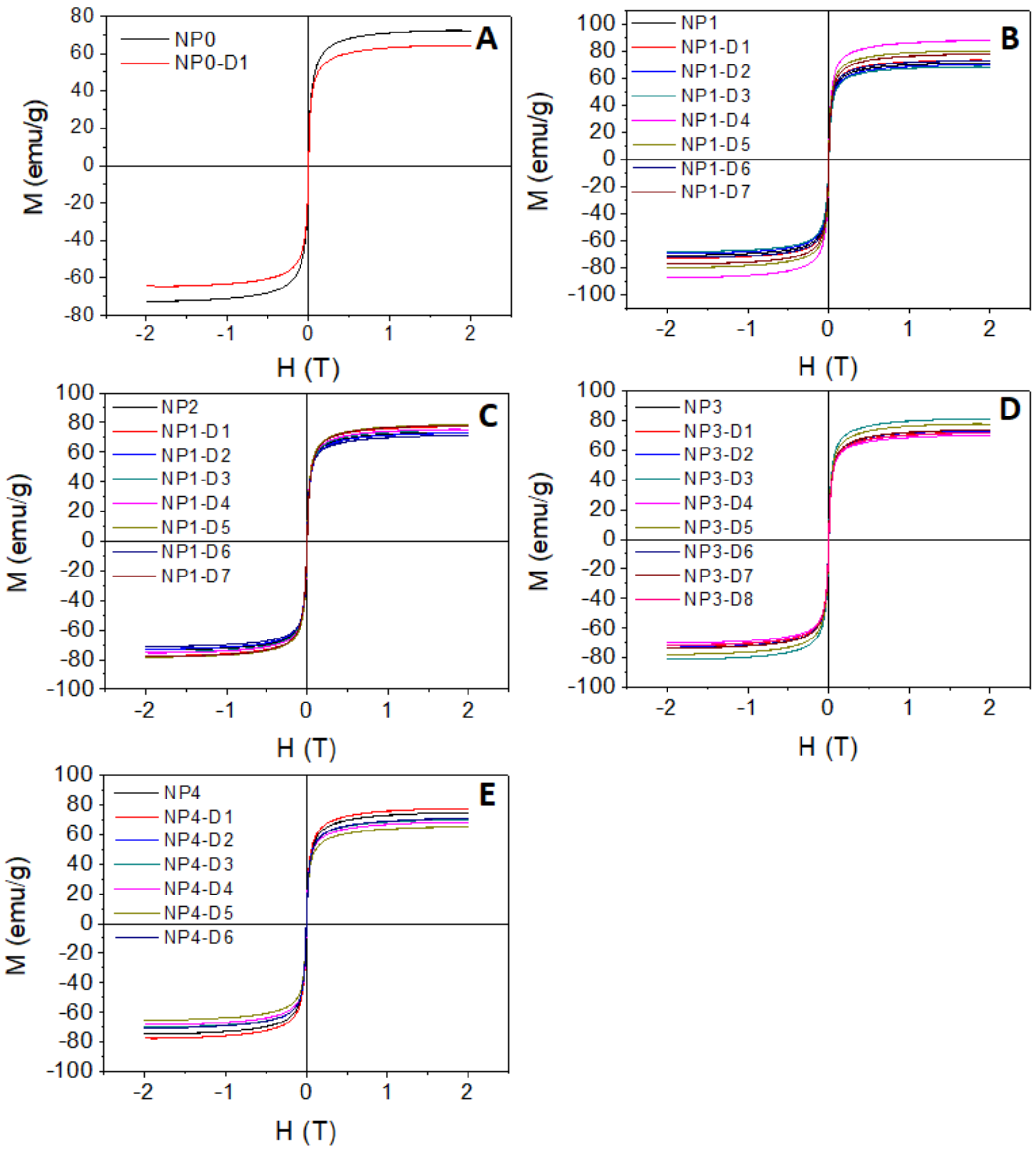

Figure S5. Hysteresis loops of dextran-coated NP0 (A), NP1 (B), NP2 (C), NP3 (D) and NP4 (E) samples.

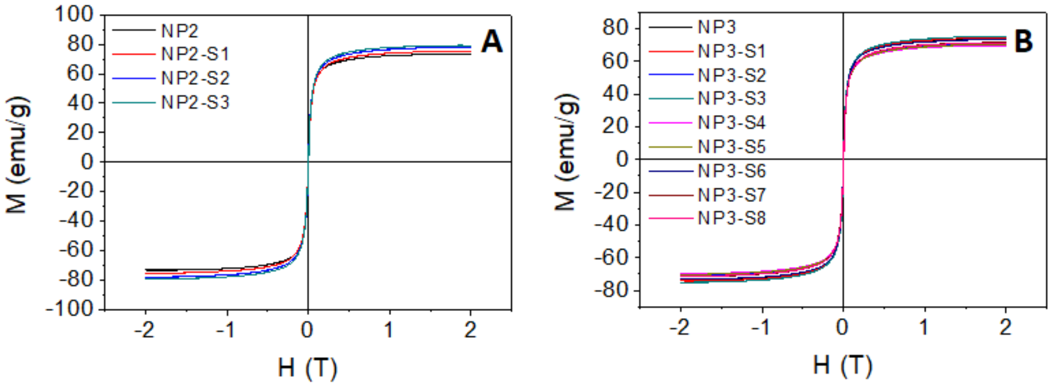

Figure S6. Hysteresis loops of starch-coated NP2 (A) and NP3 (B) samples.

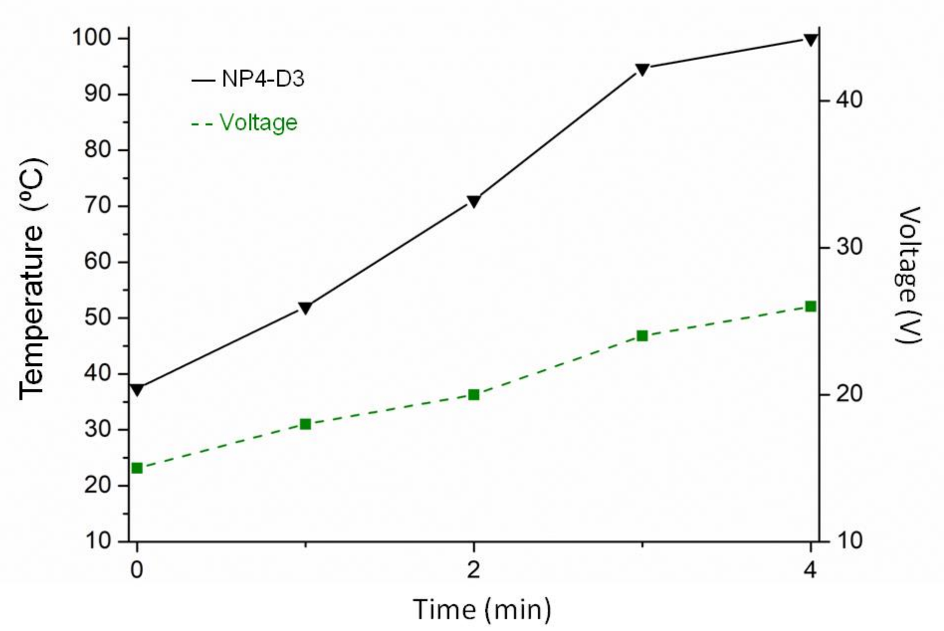

Figure S7. Temperature increase of NP4-D3 nanoparticles dispersed in water using the MACH system, increasing the voltage gradually (dotted green line).

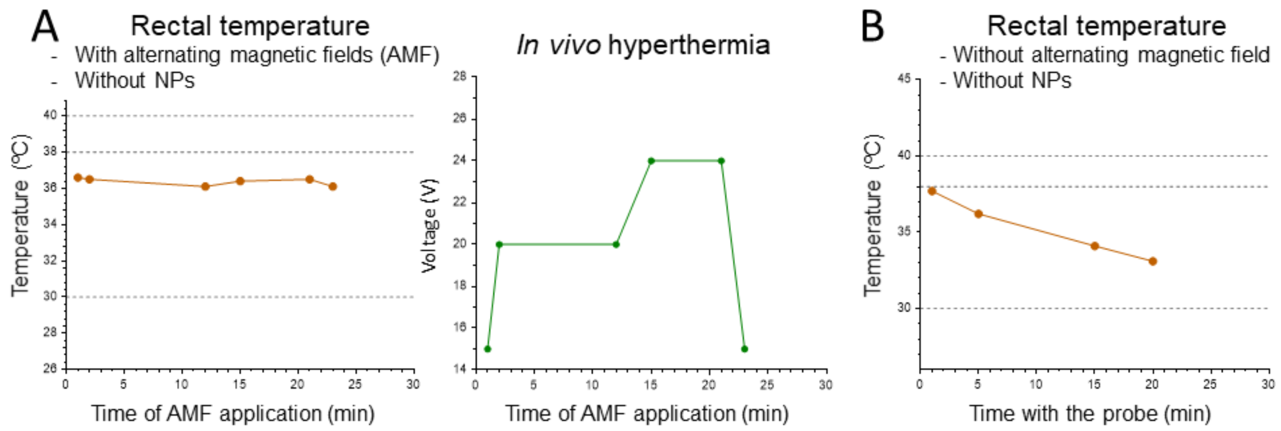

Figure S8. (A) Temperature and voltage recordings in animals with no NP inoculation. Animal without NP inoculation subjected to magnetic field of voltages raising from 15 to 20 and 24 V. No significant increase is observed in the rectal temperature (A). (B) Graph at the right corresponds to an animal that was placed within the MACH system but did not received NP nor was subjected to any magnetic field. Graph shows that in this case the rectal temperature of the animal slowly descends as a result of the anesthesia.

Table S1. Summary of the characterization data

Sample	Z-average/PdI (nm)	Surface charge (mV)	Weight loss (%)	M_S ($A{\cdot}m^2/kg$)	SAR (W/gFe) 300kHz, 4kA/m / 100kHz, 24kA/m	
**NP0**	135 (0.17)	37.0	4.3	75	-	
**NP0-D1**	114 (0.23)	7.0	56	66	10 (0.2)	58 (1)
**NP1**	138 (0.19)	33.9	3	73	-	
**NP1-D1**	93 (0.16)	-4.5	46	75	6 (0.3)	132 (0)
**NP1-D2**	136 (0.11)	0.7	47	72	3 (0.3)	111 (2)
**NP1-D3**	79 (0.18)	3.6	46	70	9 (0.2)	126 (1)
**NP1-D4**	85 (0.13)	-1.3	56	90	8 (0.2)	126 (1)
**NP1-D5**	99 (0.15)	3.8	56	82	7 (0.2)	117 (1)
**NP1-D6**	93 (0.18)	1.2	48	75	9 (0.4)	141 (2)
**NP1-D7**	135 (0.11)	0.1	47	80	4 (0.2)	121 (1)
**NP2**	141 (0.20)	39.3	5	75	-	
**NP2-D1**	102 (0.12)	2.2	51	79	6 (0.2)	146 (1)
**NP2-D2**	94 (0,14)	-1.9	51	75	8 (0.2)	156(1)
**NP2-D3**	116 (0,13)	-1.5	42	77	5 (0.1)	140 (1)
**NP2-D4**	100 (0,14)	-1.6	50	77	6 (0.4)	147 (1)
**NP2-D5**	100 (0,15)	-0.5	51	78	6 (0.4)	146 (0)
**NP2-D6**	102 (0,17)	3,2	47	73	8 (0)	163 (1)
**NP2-D7**	106 (0,15)	2.6	50	80	6 (0.3)	165 (4)
**NP2-S1**	282 (0,37)	12.3	17	77	2 (0.2)	109 (1)
**NP2-S2**	256 (0,26)	12.6	7	80	2 (0.2)	103 (3)
**NP2-S3**	229 (0,33)	10.2	9	81	2 (0.3)	103 (1)
**NP3**	166 (0.24)	39.8	4	74	-	
**NP3-D1**	104 (0.19)	1.3	52	74	7 (0.2)	146 (3)
**NP3-D2**	100 (0.19)	17.4	52	75	7 (0.1)	140 (0)
**NP3-D3**	134 (0.17)	0.4	58	83	6 (0.1)	136 (2)
**NP3-D4**	103 (0.20)	-7.4	52	72	6 (0.2)	139 (1)
**NP3-D5**	140 (0.22)	3,1	55	80	6 (0.2)	129 (1)
**NP3-D6**	122 (0,20)	-5,2	43	76	7 (0.3)	150 (0)
**NP3-D7**	121 (0,17)	-7,3	52	75	6 (0.4)	132 (0)
**NP3-D8**	111 (0,17)	-7.3	50	73	8 (0.2)	180 (2)
**NP3-S1**	152 (0.14)	12.5	9	76	2 (0.3)	124 (2)
**NP3-S2**	145 (0.19)	17.1	8	73	5 (0.2)	127 (1)
**NP3-S3**	229 (0.27)	3.3	8	77	2 (0.3)	75 (2)
**NP3-S4**	167 (0.17)	-2.2	16	72	7 (0.2)	152 (1)
**NP3-S5**	248 (0.16)	10	8	72	1 (0.2)	74 (2)
**NP3-S6**	106 (0.19)	1.5	5	75	4 (0.3)	120 (1)
**NP3-S7**	171 (0,12)	-10.8	15	73	5 (0.4)	126 (0)
**NP3-S8**	287 (0,26)	8.4	7	73	2 (0.1)	85 (2)
**NP4**	158 (0.21)	38.2	5	77	-	
**NP4-D1**	109 (0,37)	-13.2	37	80	4 (0.3)	74 (2)
**NP4-D2**	97 (0,26)	-11	31	72	7 (0.2)	123 (2)
**NP4-D3**	102 (0,22)	-8	16	72	7 (0.2)	127 (1)
**NP4-D4**	131 (0,37)	-13.2	38	70	6 (0.2)	154 (3)
**NP4-D5**	96 (0,17)	-14	36	67	6 (0.2)	156 (1)
**NP4-D6**	84 (0,21)	-1.4	35	73	7 (0.3)	154 (2)

Notes: Saturation magnetization measured at 298 K.

Abbreviations: PdI, polydispersity index ; M_S, saturation magnetization; SAR, specific absorption rate.
